# Benefits of Space Medicine Research for Healthcare on Earth

**DOI:** 10.7759/cureus.39174

**Published:** 2023-05-18

**Authors:** Bader Shirah, Hatim Bukhari, Shawna Pandya, Heba A Ezmeirlly

**Affiliations:** 1 Department of Neuroscience, King Faisal Specialist Hospital & Research Centre, Jeddah, SAU; 2 Department of Anesthesia, King Abdulaziz University Hospital, Jeddah, SAU; 3 International Institute for Astronautical Sciences Space Medicine Group, University of Alberta, Edmonton, CAN; 4 Department of Family Medicine, King Fahad Armed Forces Hospital, Jeddah, SAU

**Keywords:** earth, terrestrials, space medicine, innovations, benefits

## Abstract

Space research has brought various discoveries and benefits in the fields of health, transportation, safety measures, industry, and many more. Additionally, space research has provided a large number of discoveries and inventions in the field of medicine. Many of these inventions benefit humanity in multiple ways, especially with regard to well-being. Research objectives range from the early detection of illnesses to statistical studies that help in epidemiology. Furthermore, there are potential future opportunities that might help in the development of mankind in general and Earth medicine in particular. This review presents some of the significant inventions that were made through the journey to space and elaborate on how those inventions helped develop Earth medicine and other fields.

## Introduction and background

Space medicine is important for future orbital and interplanetary returned space flights and also contributes to the development of Earth medicine, which has developed through the discovery of new technologies on Earth but has been driven by the extreme environment and limited resources of space, and the invention of new technologies to cope with these conditions. Space technology is the general name for any technology that is related to activity in or exploration of space [1]. This review presents some of the great inventions that were made through the journey to space and elaborate on how those inventions have helped to develop Earth medicine and other fields. This article was presented at the International Astronautical Congress, IAC 2022, Paris, France, on September 18-22, 2022. Copyright was provided by International Astronautical Federation (IAF).

## Review

Remote sensing

Remote sensing involves collecting information from a distance via a sensor placed on a satellite or aircraft. Such sensors detect electromagnetic radiation (EMR) reflected from the earth up to the sensor. This EMR may originate from a passive energy source, usually the sun, or may be actively emitted by a satellite or aircraft [2].

Once these signals or EMR has been detected remotely, they are processed to determine different parameters that help interpret the Earth's surface. These parameters include radiological detection of surface temperature, sea temperature, land altitude, rainfall, humidity, air pollutants, livestock, cloud coverage, vegetation parameters, sea salinity, sea algae concentration, sea bacteria concentration, population density, and bare soil coverage. This kind of data can benefit humanity in different ways, such as identifying associations between parameters and certain diseases or disease vectors, planning rational health strategies based on previous associations to assist in disease mitigation, and direct monitoring of specific microorganisms [2,3].

One of the main uses of remote sensing is determining the association between infectious diseases and environmental parameters. For example, the associations between malaria cases and rainfall, vegetation, and surface temperature have been investigated using remotely sensed data. Based on this association, researchers have been able to predict the evolution and development of malaria, allowing them to guide public health policies. Other diseases have also been studied, including dengue fever, cholera, meningitis, and brucellosis [4]. In the context of the COVID-19 pandemic, remote sensing has also been used to monitor the way humans interact with animals, as such interactions are considered a potential source of disease outbreaks. After a long period of monitoring human-animal interactions, remotely sensed data indicated successful lockdown and quarantine procedures [5,6]. Active remote sensing has been used to detect the distinct fluorescent of cyanobacteria, which produce toxins that have been linked to medical conditions. Additionally, remote sensing can identify associations between air pollutants and non-infectious diseases. Examples of air pollutants are O_3_, NO_2_, pollens, asbestos, and wildfire smoke, which can cause respiratory diseases, coronary artery disease, premature birth, and low birth weight [2]. In other instances, remote sensing capabilities have been applied in public health, having been used to track migrant population patterns and visualize local environmental changes spurring migration events [2-6].

Global navigation satellite system

A global navigation satellite system (GNSS) comprises a group of satellites that allow individuals to locate their positions on or near the Earth. Moreover, most countries have their GNSS with their unique name. For users to determine their positions, they require an antenna that receives continuous signals coming from the satellite and a receiver that translates those signals. Once the signal is received, another system is required to display the data on a map and enable users to interpret them easily. This system is referred to as geographic information system (GIS) [2]. A GNSS is distinguished from remote sensing because it consists of multiple satellites, which gives it the ability to track moving objects. In contrast, remote sensing is generally obtained from a single satellite, forcing it to focus on a single location, although this is difficult requiring inference from the time series [5].

GNSS has been used to research various topics, including epidemiology, non-communicable diseases, physical activity of certain groups, and other topics that have contributed to the development of Earth medicine. GNSS is mainly used along with remote sensing and GIS to identify specific outcomes, causes, or possible risk factors [7]. In addition, some studies have used this space technology to consider the relationship between adolescents' use of parks and the park’s features. Moreover, other studies have targeted the physical activities of the participants by using a global positioning system (GPS) and accelerometer, enabling researchers to link the intensity of the physical activity and the location of the subjects. Interestingly, happiness has also been studied in relation to the location of subjects. Using a GPS, researchers found that individuals inhabiting natural environmental areas were happier than people living in urban areas. GNSS has also been used in tracking communicable diseases, specifically person-to-person, vector-borne, and zoonotic diseases. In addition, GNSS can help improve transportation, thereby improving public health in different ways, such as by preventing accidents and avoiding traffic jams during emergencies [2].

Satellite communication

Satellite communication refers to the ability of a satellite to transfer information from one area to another. Satellite communication has unique features such as reliable data delivery, wide-area coverage, and robustness. Satellite communication requires two main components to function: the space-based component, which is the satellite, and the Earth-based transmission, reception, and ancillary components. A typical transmission signal is sent from a station on the ground to a satellite, and the satellite then receives and amplifies the signal and sends it back to Earth [2].

Telemedicine is a valuable application of this space technology, which provides medical information or resources that are not otherwise available. Telemedicine is necessary for various situations, including remote areas or difficult-to-reach areas, and time-sensitive interventions. Moreover, telemedicine can connect medical expertise to other parties where necessary. Furthermore, telemedicine can also be used as part of a country's defense system to limit medical evacuations, thus saving significant resources [2]. Telemedicine has become particularly relevant throughout the COVID-19 global pandemic, permitting healthcare practitioners to provide virtual care while maintaining infectious disease precautions [8].

Tele-education is another important application of satellite communication, whereby new or continuing education can be provided using the same infrastructure and principles as telemedicine. Implementation of such applications can be observed in many countries, such as Japan, where 39 universities and institutions were connected via satellite communication systems [9]. Emergencies require rapid responses, whether from natural disasters, man-made emergencies, highly infectious diseases, or epidemics. The Satellite for Epidemiology (SAFE) system was developed based on satellite communication to deliver early health warnings. This system is of great value in national or international preparedness plans since the networks of common communication devices are almost always overloaded or disabled following a disaster [10].

Research and development

Physiological changes incurred in the space microgravity environment offer insight into Earth-based pathologies. Cardiovascular studies of astronauts in long-duration spaceflight have demonstrated increased insulin resistance and accelerated atherosclerosis, essentially reflecting early aging [11]. Astronauts experience muscle mass atrophy and bone density loss owing to the off-loading that occurs in microgravity despite maintaining good exercise regimes. Countermeasures looking at mitigating these effects have included genetically augmenting mice to be more resistant to muscle mass loss and bone density loss, findings that potentially can offer insight into neuromuscular disease for Earth-based populations [12]. Other astronauts experienced a decrease in their immune systems and visual functions. In addition, by providing insights into metabolic changes, protein and nucleic acid crystallization, pathway analysis, and drug efficacy comparison, the extreme environment and microgravity of space can allow for the discovery and development of new drugs [13].

Tissue chips substitute human subjects

Tissue chips are devices that can help scientists perform experiments on living cells that resemble human tissues and organs. Cells assemble and behave in microgravity differently from Earth but similar to the inside of a human body, which makes space a unique environment to perform studies and experiments. The use of tissue chips helps scientific experiments by reducing the need for animal subjects that precede clinical human trials. Unlike human subjects, tissue chips are robust and microscale, which allows sending many of these devices for a longer duration to examine the effect of the space environment for the long term. An example is the cardinal heart investigation, which used engineered heart tissues, to observe the effect of space on the heart. The experiment confirmed that space can cause changes in heart tissues. The researchers explained that the next step would be testing drugs to prevent the changes in heart tissue in space, ultimately, preventing some cardiac diseases on Earth [14].

New insights into the immune system

Astronauts experience an impaired immune system due to microgravity, which mimics the immune response that occurs when individuals age on Earth. Hence, the space environment can provide insights into age-related impairments in the immune system [15]. An experiment conducted in space illustrated that microgravity can affect the activation of T cells, which are developed from stem cells in the bone marrow, and help protect humans from infections and fight cancer. In addition, control samples were sent to space and put in a centrifuge to rule out the effect of microgravity and examine the effect of the stress of launch (sudden strong gravity) and increased radiation exposure in space. As a result, they were able to identify what microgravity can specifically do to the samples. This experiment demonstrated that there might be a resembling mechanism between the immune dysregulation of astronauts and humans on Earth. Eventually, these types of experiments can either lead to a discovery of a new drug to enhance the immune system or bring it down to Earth to treat autoimmune diseases [16].

Liquid cooling technology

In the 1960s, astronauts wore Apollo lunar suits equipped with a cooling system. This system comprised a battery-operated pump that passed cold water through the tubes lining the suit. Hence, it absorbed heat and decreased the humidity generated by the body. This technology has since evolved and has been utilized in many ways worldwide. One example is a device that delivers the optimal amount of cold and pressure to hasten the healing process of sprains of the knee, ankle, or other joints. Another spinoff of this technology is Vasper (Vasper Systems, California, United States), which combines a cooling system and vascular compression technique. In addition, Vasper can be beneficial for people performing anaerobic exercises, even for elderly or injured individuals undergoing physical therapy and people with sedentary lifestyles. It comprises cuffs connected with a liquid cooling system and a cooling vest for the chest, and the Vasper session takes approximately 30 minutes to complete. Despite the short duration of the session, it mimics the efficacy and results of exercises that are longer and more intense [17].

Adaptive cardiopulmonary resuscitation techniques

Cardiopulmonary resuscitation (CPR) is a crucial approach for victims of cardiac arrest. It is provided by placing the hands on the lower third of the sternum and applying a force sufficient to compress the chest, at least two and a half inches in adults, while also maintaining the airway and providing ventilation. However, due to the microgravity of the space environment, the rescuer is prevented from applying force on the victim's chest, making it difficult to perform effective chest compressions. Fortunately, this problem has been solved by the invention of several techniques and positional modifications that allow astronauts to perform CPR in space. One method involves securing the victim on a table-like restraint with a harness that prevents the recoil force of the chest compression from propelling the rescuer's body away from the victim. Thus, the rescuer is stabilized in place and can provide conventional CPR. Another technique for performing CPR in space includes a pneumatic vest that can be inflated rapidly enough to provide thoracic pressure resembling chest compression. However, such devices can be improved and implemented on Earth as emergency medical tools lead to a more effective and sustainable CPR technique [18].

Rehabilitation chair

Astronauts living and working in space will not experience the same amount of work and effort as felt on Earth; hence, their bones commonly start to lose density, 10 times faster than those of osteoporosis patients. Therefore, astronauts are required to perform a significant amount of exercise to limit bone density deterioration in outer space. At one point in time, researchers concluded that vibration could simulate the effect of exercise on bones leading to bone growth. They suggested that a vibration-based system that applies compressive vibratory forces could mimic weight-bearing exercises. Tests of this vibration-based system revealed a significant and beneficial impact on the muscular system as well as the skeletal system. This spinoff discovery was named VibeTech One (VibeTech Enterprises, Wisconsin, United States), which is a rehabilitation chair that uses vibrations to stimulate muscle contractions similar to weight-bearing physical exercises. This technology can help patients determine whether they are capable of exercising or have no physical strength. In addition, this technology has been used in hospitals, particularly in sports medicine, where it helps reduce muscle atrophy and keeps joints limber after an injury or surgery [17].

New postpartum hemorrhage interventions

Postpartum hemorrhage (PPH) is a maternal condition considered the leading cause of death and morbidity after childbirth. Statistically, it is responsible for 150,000 maternal deaths annually, which is considered to be a quarter of the total maternal deaths worldwide [19]. Astronauts wear inflated suits or G-suits designed to apply pressure to their bodies. This pressure helps prevent their blood from pooling in their legs, which can cause them to pass out during the extreme acceleration of take-off and when they return to Earth's stronger gravity after a spaceflight. NASA has modified the G-suit into a pressure-adjustable suit suitable for hospital settings. Moreover, NASA has implemented this new suit in a case of PPH where bleeding continued for weeks after childbirth despite all attempts to stop the bleeding, including nine surgical procedures. After only 10 hours in the modified G-suit, the bleeding stopped, and the woman started to recover. Physiologically, this technology uses an autotransfusion process that involves shunting the patient’s blood from the lower body to the upper body, where it can more easily circulate through the heart and brain. A study published in 2004 examined 14 cases of PPH in Pakistan, where this technology was used as the first line of management. The G-suits were shown to have assisted in saving the lives of 13 of these patients [20].

Remote monitoring of health conditions

Early spaceflights only lasted for short periods. Physiological changes during space travel were monitored but not transmitted back to Earth in real time. This technique was satisfactory when flights lasted for hours rather than days or months. Thus, new technologies were needed for monitoring astronauts and ensuring their safety and wellness throughout longer trips. To this end, engineers developed a new technology that monitors physiological changes in real time and transmits them to the Earth. This method is called telemetric health monitoring. This technology was later incorporated into intensive care units, where continuous vitals and other medical measurements could be transmitted, displayed, and analyzed remotely, and away from the patient. Since then, this new technology has advanced into incorporating new algorithms, which enable it to interpret data and suggest diagnoses. In addition, developers have suggested features that would allow doctors to track their patients, while they were outside of the hospital. This technology is now being adopted in next-generation smartphones and fitness trackers [21].

Point-of-care diagnostics with handheld devices

Space is a harsh environment in which all humans are vulnerable to hazardous conditions. Unfortunately, for unknown reasons, space seems to suppress the immune system in humans and makes bacteria more virulent. Hence, astronauts need reliable and fast diagnostic tests to detect health issues early. In addition, because launching heavy machinery is costly, it is impractical to send conventional laboratory equipment into space. Therefore, NASA funded Intelligent Optical Systems (IOS) to generate rapid diagnostic tests that can be launched and used in space. IOS tests use the same concept as a pregnancy test, which is a lateral flow test strip. These strips contain molecules that glow once they come into contact with bodily fluids or certain biomarkers. However, the test designed by IOS needs to be analyzed and displayed on a screen; therefore, IOS subcontracted with Cellmic (Cellmic, California, United States), which makes diagnostic hardware on smartphones. Eventually, they created a device capable of diagnosing cardiac and hepatic-related diseases based on their biomarkers. The device can now diagnose a wide variety of diseases, and its software is designed to be flexible and can be programmed based on the needs of the user. Next-generation point-of-care diagnostics include capabilities for urinalysis, renal, electrolyte, coagulation, glucose, pregnancy, and metabolic and lipid evaluation [22]. Needless to say, lightweight, portable point-of-care blood and urine diagnostics can also offer significant benefits to remote and resource-limited healthcare settings on Earth.

The need for space medicine research

Our need in the field of space medicine is crucial because it helps terrestrials in many ways, whether by providing novel methods of treatment and rehabilitation for vulnerable populations, life-saving interventions, or portable diagnostics in space. Continued advances in space medicine will continue to advance healthcare on Earth. Furthermore, space research and discoveries are beneficial not only to Earth medicine but also in other fields such as transportation, safety, the environment, and industry. In 2015, the United Nations developed a plan to help the development of humans, the planet, and prosperity known as the 2030 Agenda for Sustainable Development (2030 SDGs). Space technology can help fulfill this agenda, which contains 17 challenges that can be accomplished with the help of space technologies. Many of the challenges, such as no poverty, zero hunger, good health and well-being, and quality education, will stand to benefit through innovations in space medicine, such as food growth, pharmaceutical development, tele-education, and telehealth [23].

## Conclusions

Spaceflights and associated advances in research can lead to outstanding developments in many fields of occupation for those living on Earth, specifically in the field of health. Ongoing discoveries will continue to enable humanity to live in a healthy, peaceful, protected, and better environment. The challenges of increasingly complex, long-duration, and distant human space missions will necessitate advances in technology and research to maintain the health and longevity of future explorers, and these advances promise to offer benefits in turn for terrestrial healthcare challenges.

## References

[1] Shirah BH, Ahmed MM (2021). Patents in space medicine: an immediate call for innovations in the field. REACH.

[2] Dietrich D, Dekova R, Davy S, Fahrni G, Geissbühler A (2018). Applications of space technologies to global health: scoping review. J Med Internet Res.

[3] Sorek-Hamer M, Just AC, Kloog I (2016). Satellite remote sensing in epidemiological studies. Curr Opin Pediatr.

[4] Viana J, Santos J, Neiva R, Souza J, Duarte L, Teodoro AC, Freitas A (2017). Remote sensing in human health: a 10-year bibliometric analysis. Remote Sens.

[5] Suryaatmadja S, Maulani N (2020). Contributions of space technology to global health in the context of covid-19. JAKI.

[6] Mehmood K, Bao Y, Mushtaq S (2022). Perspectives from remote sensing to investigate the COVID-19 pandemic: a future-oriented approach. Front Public Health.

[7] Sharma P, Rattan S (2021). GIS in health: a sustenance less explored. Int J Res Publ Rev.

[8] Bhaskar S, Bradley S, Sakhamuri S (2020). Designing futuristic telemedicine using artificial intelligence and robotics in the COVID-19 era. Front Public Health.

[9] Yamauchi K, Ikeda M, Ota Y (2000). Evaluation of the Space Collaboration System: its history, image quality and effectiveness for joint case conference. Nagoya J Med Sci.

[10] Chronaki CE, Berthier A, Lleo MM (2007). A satellite infrastructure for health early warning in post-disaster health management. Stud Health Technol Inform.

[11] Afshinnekoo E, Scott RT, MacKay MJ (2020). Fundamental biological features of spaceflight: advancing the field to enable deep-space exploration. Cell.

[12] Lee SJ, Lehar A, Meir JU (2020). Targeting myostatin/activin A protects against skeletal muscle and bone loss during spaceflight. Proc Natl Acad Sci U S A.

[13] Braddock M (2020). From target identification to drug development in space: using the microgravity assist. Curr Drug Discov Technol.

[14] Mu X, He W, Rivera VA, De Alba RA, Newman DJ, Zhang YS (2022). Small tissue chips with big opportunities for space medicine. Life Sci Space Res (Amst).

[15] Crucian BE, Choukèr A, Simpson RJ (2018). Immune system dysregulation during spaceflight: potential countermeasures for deep space exploration missions. Front Immunol.

[16] (2022). National Aeronautics and Space Administration 2022. https://www.nasa.gov/sites/default/files/atoms/files/iss_benefits_for_humanity_2022_book.pdf.

[17] (2022). National Aeronautics and Space Administration 2015. https://spinoff.nasa.gov/Spinoff2015/pdf/Spinoff2015.pdf.

[18] Garshnek V (1989). Space medicine comes down to Earth. Space Policy.

[19] Sentilhes L, Merlot B, Madar H, Sztark F, Brun S, Deneux-Tharaux C (2016). Postpartum haemorrhage: prevention and treatment. Expert Rev Hematol.

[20] (2022). National Aeronautics and Space Administration 2016. https://spinoff.nasa.gov/Spinoff2016/pdf/Spinoff2016.pdf.

[21] (2022). National Aeronautics and Space Administration 2020. https://spinoff.nasa.gov/Spinoff2020/pdf/Spinoff2020.pdf.

[22] (2022). National Aeronautics and Space Administration 2017. https://spinoff.nasa.gov/Spinoff2017/pdf/Spinoff2017.pdf.

[23] (2022). United Nations General Assembly: Revised draft “Space2030” agenda and implementation plan. https://www.unoosa.org/res/oosadoc/data/documents/2020/aac_105c_2l/aac_105c_2l_316_0_html/AC105_C2_L316E.pdf.

